# Surgical treatment of colonic Crohn’s disease: a national snapshot study

**DOI:** 10.1007/s00423-020-02038-z

**Published:** 2020-12-02

**Authors:** SICCR Current status of Crohn’s disease surgery collaborative, Valerio Celentano, Gianluca Pellino, Matteo Rottoli, Gilberto Poggioli, Giuseppe Sica, Mariano Cesare Giglio, Michela Campanelli, Claudio Coco, Gianluca Rizzo, Francesco Sionne, Francesco Colombo, Gianluca Sampietro, Giulia Lamperti, Diego Foschi, Ferdinando Ficari, Ludovica Vacca, Marta Cricchio, Francesco Giudici, Lucio Selvaggi, Guido Sciaudone, Roberto Peltrini, Andrea Manfreda, Luigi Bucci, Raffaele Galleano, Omar Ghazouani, Luigi Zorcolo, Simona Deidda, Angelo Restivo, Andrea Braini, Francesca Di Candido, Matteo Sacchi, Michele Carvello, Stefania Martorana, Giovanni Bordignon, Imerio Angriman, Angela Variola, Giuliano Barugola, Mirko Di Ruscio, Marta Tanzanu, Andrea Geccherle, Francesca Paola Tropeano, Gaetano Luglio, Diego Sasia, Marco Migliore, Maria Carmela Giuffrida, Enrico Marrano, Gianluigi Moretto, Harmony Impellizzeri, Gaetano Gallo, Giuseppina Vescio, Giuseppe Sammarco, Giovanni Terrosu, Giacomo Calini, Andrea Bondurri, Anna Maffioli, Gloria Zaffaroni, Andrea Resegotti, Massimiliano Mistrangelo, Marco Ettore Allaix, Fiorenzo Botti, Matteo Prati, Luigi Boni, Serena Perotti, Michela Mineccia, Antonio Giuliani, Lucia Romano, Giorgio Maria Paolo Graziano, Luigi Pugliese, Andrea Pietrabissa, Gian Gaetano Delaini, Antonino Spinelli, Francesco Selvaggi

**Affiliations:** Queen Alexandra Hospital Portsmouth Hospitals NHS Trust, Portsmouth, PO6 3LY UK

**Keywords:** Crohn’s disease, Crohn’s colitis, Inflammatory bowel disease, Segmental colectomy, Proctocolectomy, National audit

## Abstract

**Aim:**

The different surgical options for patients with colonic Crohn’s disease (CD) include segmental colectomy, subtotal colectomy or proctocolectomy with end ileostomy. We present a national, multicentre study, promoted by the Italian Society of Colorectal Surgery with the aim to collect benchmark data and national variations on multidisciplinary management and postoperative outcomes of patients undergoing surgery for colonic CD.

**Methods:**

All adult patients having elective surgery for colonic CD from June 2018 to May 2019 were eligible for participation in this retrospective study. The primary outcome measure was postoperative morbidity within 30 days of surgery.

**Results:**

One hundred twenty-two patients were included: 55 subtotal colectomy, 30 segmental colectomy, 25 proctectomy and 12 proctocolectomy. Eighty-six patients (70.4%) were discussed at the inflammatory bowel disease (IBD) multidisciplinary team meeting (MDT) prior to surgery. This ranged from 76.6% for segmental colectomy to 60% for subtotal colectomy, 66.6% for proctocolectomy and 48% for proctectomy. The proportion of patients counselled by a stoma nurse preoperatively was 50%. Laparoscopy was associated with reduced postoperative morbidity (*p* = 0.017) and shorter length of hospital stay (*p* < 0.001), whilst pre-operative anti-TNF was associated with Dindo-Clavien ≥ 3 complications (*p* = 0.023) and longer in-hospital stay (*p* = 0.007). The main procedure performed (segmental colectomy, subtotal colectomy, proctocolectomy or proctectomy) was not associated with postoperative morbidity (*p* = 0.626).

**Conclusions:**

Surgery for colonic CD has a high rate of postoperative complications. Almost a third of the patients were not preoperatively discussed at the IBD MDT, whilst the use of minimally invasive surgery for surgical treatment of colonic CD ranges from 40 to 66%.

**Supplementary Information:**

The online version contains supplementary material available at 10.1007/s00423-020-02038-z.

## Introduction

Crohn’s disease (CD) is a chronic inflammatory condition that can affect any part of the gastrointestinal system, with one third of the patients having the disease confined to the large bowel [1]. There are different surgical options for patients with colonic CD, including segmental colectomy, subtotal colectomy with ileorectal anastomosis or total proctocolectomy with end ileostomy [2]. Surgical treatment of colonic CD can be challenging in view of the high risk of postoperative septic complications [3], the high rate of clinical and surgical recurrence and the impaired functional outcomes and quality of life that can result following extensive surgery. For this particular presentation of CD, a multidisciplinary management is of outmost importance [4], because evidence concerning the ideal surgical strategy is scarce. Moreover, differently from ulcerative colitis, surgical treatment of colonic CD comprises less extensive alternatives to proctocolectomy.

The most recent guidance on surgical treatment of inflammatory bowel diseases (IBD) of the Association of Coloproctologists of Great Britain and Ireland (ACPGBI) suggested that a segmental or subtotal colectomy and ileorectal anastomosis are both viable bowel-preserving options in case of isolated segmental colonic CD [5]. A recent meta-analysis concluded that segmental, subtotal and proctocolectomy can be equally effective in patients with colonic CD, but the quality of the included studies limits the external validity of the findings [6].

Of note, most studies do not consistently record key performance indicators of CD surgery, with paucity of audits on patient-reported outcome measures (PROMs) [7]. A limited number of studies have focused on postoperative complication in patients with colonic CD [8] with some being small case series over long period of times, or even before the availability of treatments with biologics [9, 10]. The aim of this study was to describe the management of colonic CD according to several quality indicators of postoperative outcome in patients included in a nationwide study promoted by the Italian Society of Colorectal Surgery (SICCR).

## Methods

### Study settings

In 2019, the Italian Society of Colorectal Surgery (SICCR) designed the retrospective, multicentre, snapshot study “Current Status of Crohn’s disease surgery”. The present STROBE [11] compliant study evaluates the treatment of colonic CD in Italy. Details have been previously reported [12]. Briefly, after developing the study protocol, which was approved by the SICCR research board, the steering committee invited Italian colorectal units to join the initiative via an open call and newsletters.

### Ethical statement

Each local principal investigator (PI) was responsible for approval at the local ethical committee, and all participating centres obtained ethical approval. Informed consent was deemed not necessary (retrospective study) from the ethics committees.

### Inclusion and exclusion criteria

All patients (aged 16 or older), who underwent elective surgery for colonic CD from 1 June 2018 to 31 May 2019, were evaluated. The following procedures were included: segmental colectomy, subtotal colectomy, proctocolectomy and proctectomy. If a right hemicolectomy was performed in the context of CD terminal ileitis this was excluded, as were patients having a concomitant segmental left colonic resection in the setting of penetrating ileitis. Patients having surgery for cancer, primary or recurrent CD of the distal ileum were excluded. The data collection period of 12 months was decided by the steering committee in order to collect a snapshot on the current status of CD surgery in Italy, to guide targeted quality improvement, where indicated. Patients were considered to have been treated with tumor necrosis factor (TNF)-alpha inhibitors if they had received infliximab infusion within the 4 weeks prior to surgery or adalimumab injection within 2 weeks prior the surgery. Immunosuppressor use was defined as azathioprine or methotrexate within 2 weeks from surgery, whilst an equivalent dose of 20 mg or more of prednisolone within one week of surgery was defined as steroids use.

### Study endpoints and outcome measures

The primary endpoint consisted of postoperative morbidity within 30 days of surgery. Postoperative surgical site infections (SSIs) and use of laparoscopy were the secondary outcome measures.

### Data collection and definitions

Collected data included baseline information and demographics, Montreal classification, preoperative medical treatment, indication for surgery, American Society of Anaesthesiologists (ASA) grade and operative details.

The following were used as key performance indicators:

- Stoma rate, defined as the percentage of patients who received an intestinal stoma (both planned and unplanned, both temporary and definitive);
- Surgical access and conversion rate;
- Length of hospital stay (LOS), defined as duration of the stay from day of surgery to discharge;
- 30-day postoperative morbidity, as any complication occurring during the hospital stay or within 30 days of surgery;
- SSIs within 30 days of surgery, defined as any superficial or deep septic complication related to the surgical site;
- Readmissions and reoperations within 30 days from discharge;

Data on use of PROMs was also collected, as well as preoperative discussion in a dedicated multidisciplinary team meeting (MDT), preoperative consultation with a stoma care nurse and use of total parenteral nutrition (TPN). Data collection was responsibility of the local PI and was performed via the use of prospectively maintained databases, or retrieved using hospital coding registries.

### Statistical analysis

Categorical variables are presented as frequency and percentages and were compared using the chi-square test or Fisher’s exact test, as appropriate. Continuous variables are presented as mean (± standard deviation) or median (range) according to their distribution and were compared with the use of Student’s *t* test or the Mann-Whitney *U* test in case of normal or skewed distribution, respectively. Uni- and multivariable logistic regression analyses were run in order to identify variables associated with binary outcomes. Clinically relevant variables with a *p* value equal to 0.10 or less at the univariate analysis were included in the multiple regression model. The odds ratio (ORs) with a 95% confidence interval (CI) was estimated as measure of association. All reported *p* values were two-tailed, and *p* values of less than 0.05 were considered to be statistically significant. Statistical analysis was performed by using IBM SPSS Statistics for Windows, version 25.0 (IBM Corp., Armonk, NY, USA).

## Results

**Fig. 1:**
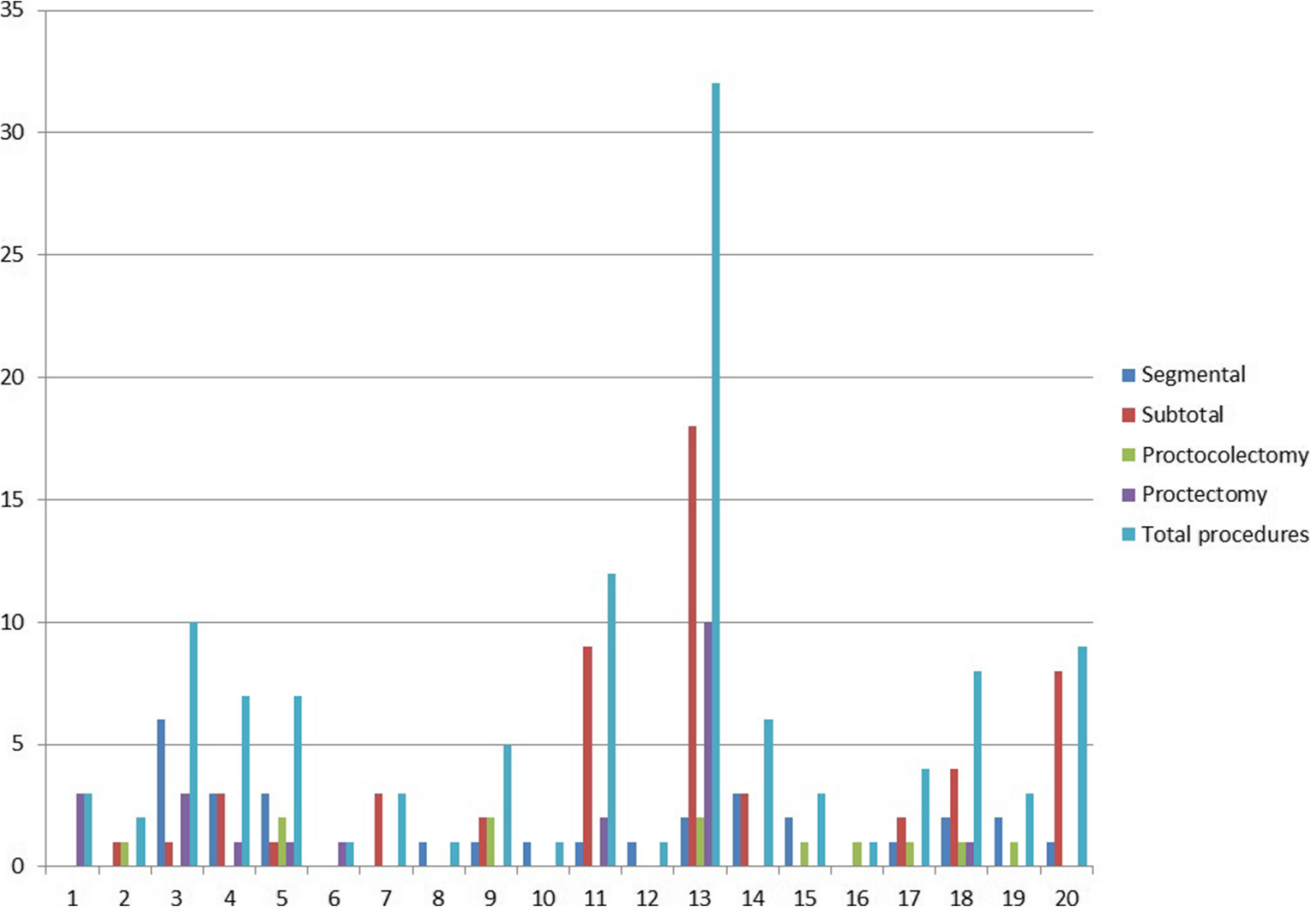
Number of surgical resections performed for colonic Crohn’s disease in the 20 participating hospitals

Overall, 144 patients treated in 20 hospitals were evaluated for inclusion. After excluding 17 patients undergoing urgent or emergency surgery and five patients who had ileostomy formation only, 122 patients were included in the analysis. Out of the included patients, 55 patients underwent subtotal colectomy (45.1%), 30 segmental colectomy (24.6%), 25 proctectomy (20.5%) and 12 proctocolectomy (9.8%). The procedures performed in the segmental colectomy group were as follows: 19 left hemicolectomies, 1 transverse colectomy, 5 Hartmann’s procedures and 5 anterior resections. In the subtotal colectomy group, 21 patients received an ileorectal anastomosis (38.2%). Every hospital was allocated a unique identifier number (ID). The number of total patients per hospital ranged from one to 32, as shown in Fig. 1. Baseline patients’ characteristics and preoperative medical treatments are detailed in Table 1.

**Table 1:** Characteristics of the included patients

	Segmental colectomy	Subtotal colectomy	Proctocolectomy	Proctectomy	*p*
Patients	30	55	12	25	
M:F	14:16	23:32	7:5	12:13	0.761
Age	47 (28–79)	39 (17–83)	50 (24–76)	47 (22–77)	0.163^†^
BMI	22 (16–27)	21 (14–30)	24 (15.4–30)	24.5 (18–38)	0.006^†^
Recurrent disease	15 (50%)	24 (43.6%)	8 (66.6%)	20 (80%)	0.017
Montreal classification A	A1 11 (36.7%) A2 12 (40%) A3 7 (23.3%)	A1 11 (20%) A2 27 (49.1%) A3 17 (30.9%)	A1 2 (16.6%) A2 2 (16.6%) A3 8 (66.6%)	A1 1 (4%) A2 8 (8%) A3 14 (56%)	0.009
Montreal classification L	L2 19 (63.3%) L3 11 (36.7%)	L2 30 (54.5%) L3 25 (45.5%)	L2 4 (33.3%) L3 8 (66.6%)	L2 17(68%) L3 8 (32%)	0.136
Montreal classification B	B1 3 (10%) B2 18 (60%) B3 9 (30%)	B1 17 (30.9%) B2 25 (45.5%) B3 13 (23.6%)	B1 3 (25%) B2 5 (41.7%) B3 4 (33.3%)	B1 10 (40%) B2 7 (28%) B3 8 (32%)	0.429
Perianal disease	16 (53.3%)	23 (41.8%)	8 (66.6%)	18 (72%)	0.231
Previous abdominal surgery	16 (53.3%)	29 (52.7%)	6 (50%)	24 (96%)	0.001
MDT	23 (76.6%)	33 (60%)	8 (66.6%)	12 (48%)	0.169
USS	17 (56.6%)	29 (52.7%)	4 (33.3%)	8 (32%)	0.173
MRI	21 (70%)	35 (63.6%)	7 (58.3%)	19 (76%)	0.538
CT	24 (80%)	23 (41.8%)	7 (58.3%)	8 (32%)	<0.001
Capsule	0	1 (1.8%)	0	0	0.746
Stoma nurse	15 (50%)	22 (40%)	6 (50%)	9 (36%)	0.674
Dietitian	15 (50%)	17 (30.9%)	2 (16.6%)	2 (8%)	0.042
TPN	7 (23.3%)	8 (14.5%)	2 (16.6%)	2 (8%)	0.433
Steroids	11 (36.6%)	27 (49.1%)	4 (33.3%)	4 (16%)	0.043
Immunosupp	3 (10%)	7 (12.7%)	2 (16.6%)	2 (8%)	0.858
Anti-TNF	9 (30%)	16 (29.1%)	0	1 (4%)	0.010

*M* male, *F* female, *BMI* body mass index, *Montreal classification A* age at diagnosis, *L* location, *B* phenotype, *MDT* multidisciplinary team; *USS* ultrasound, *MRI* magnetic resonance imaging, *CT* computed tomography, *TPN* total parenteral nutrition, *TNF* tumor necrosis factor

*p* values are from Pearson’s chi square and from ^†^one-way ANOVA

### Multidisciplinary management and preoperative medical treatment

Eighty-six patients (70.4%) were discussed at the IBD MDT prior to surgery. This ranged from 76.6% for segmental colectomy to 60% for subtotal colectomy (*p* = 0.18), 66.6% for proctocolectomy (*p* = 0.62) and 48% for completion proctectomy (*p* = 0.04).

The proportion of patients counselled by a stoma nurse preoperatively was 50% in the segmental colectomy group, compared to 40% and 50% of subtotal colectomies and proctocolectomies, respectively. A higher number of patients were assessed by a dietitian and received preoperative TPN in the segmental colectomy group, as shown in Table 1.

### Postoperative morbidity and length of stay

No 30-days post-operative mortality was reported. Overall morbidity within 30 days of surgery is shown in Table 2. Severe complications (Clavien-Dindo ≥ III), reoperations and readmissions were slightly more common following segmental colectomy compared to subtotal colectomy (Table 2) but this was not statistically significant. LOS ranged from 8 to 10 days (median 8.5 days, interquartile range 7–13). SSIs were the most common complications, with an incidence ranging from 8 to 27.3%.

**Table 2:** Surgical outcomes

	Segmental colectomy	Subtotal colectomy	Proctocolectomy	Proctectomy	*p*
Patients	30	55	12	25	
Stoma made	20 (66.6%)	36 (65.4%)	12 (100%)	25 (100%)	0.004
Lap approach	19 (63.3%)	34 (61.8%)	8 (66.6%)	11 (44%)	0.586
Conversion	3 (15.8%)	7 (20.6%)	2 (25%)	0	0.205
LOS	10 (4–34)	8 (4–66)	10 (4–26)	9 (2–34)	0.750^†^
Morbidity	11 (36.6%)	23 (41.8%)	5 (41.6%)	9 (36%)	0.416
SSIs	7 (23.3%)	15 (27.3%)	3 (25%)	2 (8%)	0.281
Dindo ≥ 3	4 (13.3%)	4 (7.3%)	1 (8.3%)	2 (8%)	0.902
Reoperations	4 (13.3%)	2 (3.6%)	1 (8.3%)	2 (8%)	0.055
Readmissions	4 (13.3%)	1 (1.8%)	0	0	0.520

*LOS* length of stay, *SSIs* surgical site infections, Lap laparoscopy

^†^*p* from one-way ANOVA

A subgroup analysis of the patients receiving an anastomosis without diversion between the segmental colectomy group (10 patients) and subtotal colectomy with ileorectal anastomosis group (19 patients) was performed. This revealed that 5 (50%) and 8 (42.1%) patients developed postoperative complications in the segmental colectomy and subtotal colectomy group respectively (*p* = 0.68), whilst severe complications (Clavien-Dindo ≥ III) occurred in 3 (30%) compared to 1 (5.2%) patients in the two groups (*p* = 0.07).

### Laparoscopic surgery and conversion rate

There was no difference in the use of laparoscopic surgery between the included procedures (Table 2). There were no conversions to open surgery in patients undergoing completion proctectomy, whilst for segmental colectomies, subtotal colectomies and proctocolectomies, the conversion rate ranged from 15.8 to 25%.

### Patient-reported outcome measures (PROMs)

There was a low adoption of standardised questionnaires to assess quality of life, bowel, urinary and sexual function following surgery for colonic CD, as these assessments were performed only in 5 patients (4%).

### Univariate and multivariate analysis

At multivariate analysis, a laparoscopic approach (Table 3) was associated with reduced postoperative morbidity (*p* = 0.017) and shorter LOS (*p* < 0.001), whilst pre-operative administration of anti-TNF correlated with Dindo-Clavien ≥ III postoperative complications (*p* = 0.023) and longer LOS (*p* = 0.007). The use of preoperative TPN and anti-TNF was associated with postoperative SSIs at univariate analysis (Table 4). Surgery for recurrent CD was associated with conversion from a laparoscopic to an open approach (*p* = 0.029) and to SSIs (*p* = 0.042). The main procedure performed (segmental colectomy, subtotal colectomy, proctocolectomy or proctectomy) was not associated with postoperative morbidity (*p* = 0.626) or SSIs (*p* = 0.322).

**Table 3:** Univariate and multivariate analysis for postoperative morbidity in patients with colonic Crohn’s disease

Postoperative morbidity						
	Univariate analysis			Multivariate analysis		
Variable	OR	95% CI	*p*	OR	95% CI	*p*
Age	1.016	0.992–1.04	0.193			
Sex (female)	0.663	0.318–1.375	*0.271*			
BMI	0.948	0.866–1.032	0.229			
ASA grade ≥ 3	1.883	0.808–4.425	0.142			
Recurrent CD	1.256	0.605–2.635	0.542			
Main procedure	1.072	0.812–1.419	0.626			
Montreal B = 3	2.045	0.919–4.591	0.080	1.357	0.527–3.490	0.527
Montreal L = 3	0.560	0.259–1.186	0.134			
Perianal disease	0.927	0.454–1.888	0.834			
Preoperative steroids	0.794	0.368–1.68	0.551			
Preoperative immunosuppression	3.138	1.011–10.838	0.054	2.825	0.652–12.246	0.165
Preoperative anti-TNF	3.150	1.302–7.952	0.012	2.967	0.944–9.323	0.063
Preoperative TPN	1.127	0.405–3.029	0.813			
Access (minimally invasive)	0.382	0.180–0.809	0.012	0.344	0.144–0.826	**0.017**

*BMI* body mass index, *ASA* American Society of Anaesthesiologists, *OR* odds ratio, *CI* confidence interval, *TNF* tumor necrosis factor, *TPN* total parenteral nutrition

**Table 4:** Univariate and multivariate analysis for postoperative surgical site infections (SSIs) in patients with colonic Crohn’s disease

Postoperative SSIs						
	Univariate analysis			Multivariate analysis		
Variable	OR	95% CI	*p*	OR	95% CI	*p*
Age	0.99	0.97–1.02	0.613			
Sex (female)	1.31	0.55–3.18	0.543			
BMI	1.00	0.91–1.10	0.959			
ASA grade ≥ 3	1.44	0.53–3.69	0.453			
Recurrent CD	2.92	1.17–8.03	0.027	3.07	1.04–9.05	0.042
Main procedure	0.85	0.61–1.17	0.322			
Montreal B = 3	1.98	0.79–4.84	0.137			
Montreal L = 3	1.34	0.56–3.19	0.501			
Perianal disease	1.01	0.44–2.34	0.983			
Preoperative steroids	0.92	0.37–2.19	0.844			
Preoperative immunosuppression	4.35	1.35–14.12	0.013	2.06	0.48–8.92	0.333
Preoperative anti-TNF	3.62	1.40–9.38	0.008	2.55	0.76–8.54	0.129
Preoperative TPN	4.20	1.48–11.96	0.007	2.95	0.91–9.60	0.072
Access (minimally invasive)	0.66	0.28–1.55	0.339			

*BMI* body mass index, *ASA* American Society of Anaesthesiologists, *OR* odds ratio, *CI* confidence interval, *TNF* tumor necrosis factor, *TPN* total parenteral nutrition

## Discussion

Our study confirms that surgery for colonic CD is affected by high postoperative morbidity, ranging from 36 to 41.8%, with SSIs representing the most frequent complication. Our study reported that approximately one-third of the patients undergoing segmental colectomy were on anti-TNF treatment, whilst up to half of the subtotal colectomies were currently on steroids. We found that anti-TNF treatment was associated with longer LOS and with severe postoperative morbidity (Dindo-Clavien ≥ 3), likely reflecting a selected group of patients compromised or with more complex disease, adding to the literature debate on the effect of biologics on surgical morbidity [13, 14]. Conversely, we did not demonstrate an increased complication rate for segmental resection compared to subtotal colectomy and proctocolectomy, which is considered to be up to a 2-fold increase as reported in a recent meta-analysis [6]. It is concerning that almost 30% of the patients did not receive preoperative input from the wider IBD MDT, with preoperative discussion ranging from 76% for segmental colectomies to 40% for proctectomies. Moreover, as surgical stoma was a common outcome of surgery for colonic CD (it was performed in 93 patients, 76.2%), it should be mandatory for patients to be counselled preoperatively by a dedicated stoma care team, whilst this only took place for 36 to 50% of the patients. Similarly, preoperative optimisation with TPN and input from the dietitians was more frequent in patients undergoing segmental resections. Prompt planning of elective surgery and high volume IBD surgeons may impact on bowel sparing surgery, preoperative medical treatment and postoperative outcomes when surgery is performed in specialist referral centres, with support from dedicated teams. Rather than expressing a particular preference for one surgical approach over the others, our results highlight the need for careful patient selection, preoperative optimisation and multidisciplinary input, to individualise the treatment according to extent of disease but also patients’ expectations and performance status, considering the high risk of postoperative complications and the known high recurrence rate of up to 65% for segmental resections [15].

We collected data on patients undergoing surgery during a 12 months period between 2018 and 2019 obtaining an up to date snapshot of the current status of surgery for colonic CD in Italy. Our results confirm the advantages of a minimally invasive approach for surgical treatment of colonic CD in terms of reduced postoperative morbidity and shorted LOS. However, we found a relatively low use of laparoscopic surgery, which ranged from 44 to 66% with a considerable conversion rate up to 25%.

Our study demonstrated significant heterogeneity across the 20 participating Italian hospitals in the number of cases performed per year, with 11 hospitals (55%) performing less than five procedures during the 12 months recruitment period. Rectal resection in CD can be particularly difficult in view of adhesions, inflammation and chronic pelvic abscesses, with possible implications on postoperative functional outcomes such as genitourinary function [16]. Of the 16 hospitals performing at least one procedure between completion proctectomy and proctocolectomy, only four (25%) performed at least three procedures per year, which could also reflect on the difficulties for surgeons in training to accumulate exposure to these complex procedures [17]. To reduce this variability, the SICCR recently published a national position statement with the aim to standardise multidisciplinary management and surgical treatment of CD nationally [18], and guidelines of several international societies have been released to optimize CD outcomes (e.g. European Crohn’s Colitis Organisation—ECCO [19], the American Society of Colorectal Surgery—ASCRS) [20]. A general aim of guidelines is to reduce variations in practice, by providing evidence based guidance on the best treatment options, with the aim to enhance patients’ outcomes.

Our study is limited by the small sample size, which may have underpowered the statistical analysis, and retrospective design with an intrinsic risk of recall and information bias. Many of the participating centres only included a small number of patients and SSIs and complication rates were directly reported by the operating surgeon, with variability in definitions [21] and high likelihood of selection bias. Moreover, our study focused on short term outcomes of surgery for colonic CD, without evaluating long term surgical recurrence, which should be the focus of prospective studies including mandatory PROMs evaluation.

## Conclusions

Surgery for CD of the colon has a high rate of postoperative complications, which was associated with preoperative anti-TNF treatment in our study. Up to 30% of the patients with colonic CD did not receive input from the IBD MDT, whilst the use of minimally invasive surgery for surgical treatment of colonic CD ranges from 40 to 66% despite being beneficial for patients with a shorter LOS and reduced morbidity.

## Supplementary Information

ESM 1 (DOCX 19 kb)

ESM 2 (DOCX 14 kb)
