# Correction to: Surgical treatment of colonic Crohn’s disease: a national snapshot study

**DOI:** 10.1007/s00423-021-02119-7

**Published:** 2021-03-02

**Authors:** SICCR current status of Crohn’s disease surgery collaborative, Valerio Celentano, Gianluca Pellino, Matteo Rottoli, Gilberto Poggioli, Giuseppe Sica, Mariano Cesare Giglio, Michela Campanelli, Claudio Coco, Gianluca Rizzo, Francesco Sionne, Francesco Colombo, Gianluca Sampietro, Giulia Lamperti, Diego Foschi, Ferdinando Ficari, Ludovica Vacca, Marta Cricchio, Francesco Giudici, Lucio Selvaggi, Guido Sciaudone, Roberto Peltrini, Andrea Manfreda, Luigi Bucci, Raffaele Galleano, Omar Ghazouani, Luigi Zorcolo, Simona Deidda, Angelo Restivo, Andrea Braini, Francesca Di Candido, Matteo Sacchi, Michele Carvello, Stefania Martorana, Giovanni Bordignon, Imerio Angriman, Angela Variola, Giuliano Barugola, Mirko Di Ruscio, Marta Tanzanu, Andrea Geccherle, Francesca Paola Tropeano, Gaetano Luglio, Diego Sasia, Marco Migliore, Maria Carmela Giuffrida, Enrico Marrano, Gianluigi Moretto, Harmony Impellizzeri, Gaetano Gallo, Giuseppina Vescio, Giuseppe Sammarco, Giovanni Terrosu, Giacomo Calini, Andrea Bondurri, Anna Maffioli, Gloria Zaffaroni, Andrea Resegotti, Massimiliano Mistrangelo, Marco Ettore Allaix, Fiorenzo Botti, Matteo Prati, Luigi Boni, Serena Perotti, Michela Mineccia, Antonio Giuliani, Lucia Romano, Giorgio Maria Paolo Graziano, Luigi Pugliese, Andrea Pietrabissa, GianGaetano Delaini, Antonino Spinelli, Francesco Selvaggi

**Affiliations:** 1 Portsmouth Hospitals NHS Trust, Portsmouth, UK; 2 University of Portsmouth, Portsmouth, UK; 3 Department of Surgery and Cancer, Imperial College, London, UK; 4 Department of Advanced Medical and Surgical Science, Universita’ degli Studi della Campania Luigi Vanvitelli, Naples, Italy; 5 Surgery of the Alimentary Tract, Sant’Orsola Hospital, Department of Medical and Surgical Sciences, Alma Mater Studiorum University of Bologna, Bologna, Italy; 6 Minimally Invasive & Gastro-Intestinal Surgical Unit, Department of Surgery, Policlinico Tor Vergata, Rome, Italy; 7 Department of Clinical Medicine and Surgery, Federico II University of Naples, Naples, Italy; 8 U.O.C. Chirurgia Generale 2 - Fondazione Policlinico Universitario “Agostino Gemelli” IRCCS, Università Cattolica del Sacro Cuore, Rome, Italy; 9 General Surgery Unit, Department of Biomedical and Clinical Sciences “L. Sacco”, University of Milan, ASST Fatebenefratelli Sacco, Milan, Italy; 10 Division of General and HPB Surgery, Department of Surgery, ASST Rhodense - Rho Memorial Hospital, 20017 Rho, Milan Italy; 11 IBD Unit, Careggi University Hospital, Florence, Italy; 12 University of Naples Federico II, Naples, Italy; 13 Santa Corona Hospital, Pietra Ligure, Italy; 14 Colon and Rectal Surgery Unit, University of Cagliari, Cagliari, Italy; 15 Friuli Occidentale Hospital, Pordenone, Italy; 16 Division of Colon and Rectal Surgery, Humanitas Clinical and Research Center IRCCS, Via Manzoni 56, 20089 Rozzano, Milano Italy; 17 Department of Surgical Oncological and Gastroenterological Sciences University of Padova, Surgical Unit, Padova, Italy; 18 IRCCS “Sacro Cuore - Don Calabria”, Negrar, Vr Italy; 19 Department of Public Health, University of Naples Federico II, Naples, Italy; 20 Department of Surgery, Santa Croce e Carle Hospital, Cuneo, Italy; 21 Department of Surgery, “Pederzoli” Hospital, Peschiera del Garda, Verona, Italy; 22 Department of Medical and Surgical Sciences, University of Catanzaro, Catanzaro, Italy; 23 Department of Health sciences, University of Catanzaro, Catanzaro, Italy; 24 Department of Surgery, University Hospital “Santa Maria della Misericordia”, Udine, Italy; 25 Unit 1, General Surgery, Luigi Sacco University Hospital, Milan, Italy; 26 Department of Surgical Sciences, Citta della Salute e della Scienza di Torino, Presidio Molinette, University Hospital, Turin, Italy; 27 Department of General Surgery, Fondazione IRCCS Ca’ Granda Ospedale Maggiore Policlinico, Milan, Italy; 28 Division of General and Oncologic Surgery, Mauriziano Hospital, Turin, Italy; 29 San Salvatore Hospital. Department of Biotechnological and Applied Clinical Sciences, University of L’Aquila, L’Aquila, Italy; 30 Fondazione IRCCS Policlinico San Matteo di Pavia, Università degli Studi di Pavia, Pavia, Italy; 31 Department of Biomedical Sciences, Humanitas University, Via Rita Levi Montalcini 4, 20090 Pieve Emanuele, Milano Italy

**Correction to: Langenbeck’s Archives of Surgery (2020)**

10.1007/s00423-020-02038-z

The original version of this article unfortunately contained a mistake in the list of contributors. The 72 collaborators’ names were not tagged in the published version of the article.

The 72 collaborators’ names can be found below and in the online version of the article.

Valerio Celentano^1,2,3^, Principal investigator. Conception and design of the study, analysis and interpretation of data, drafting the article, critical review of the article, and study steering committee

Gianluca Pellino^4^, Conception and design of the study, analysis and interpretation of data, drafting the article, critical review of the article, and study steering committee

Matteo Rottoli^5,^ Local principal investigator, acquisition of data, analysis and interpretation of data, and critical review of the article

Gilberto Poggioli^5^, Data collection and analysis and draft manuscript review

Giuseppe Sica^6^, Data collection and analysis and draft manuscript review

Mariano Cesare Giglio^7^, Statistical analysis and draft manuscript

Michela Campanelli^6^, Data collection and analysis and draft manuscript review

Claudio Coco^8^, Local principal investigator, acquisition of data, analysis and interpretation of data, and critical review of the article

Gianluca Rizzo^8^, Data collection and analysis and draft manuscript review

Francesco Sionne^8^, Data collection and analysis and draft manuscript review

Francesco Colombo^9^, Local principal investigator, acquisition of data, analysis and interpretation of data, and critical review of the article

Gianluca Sampietro^10^, Local principal investigator, data analysis, and draft manuscript review

Giulia Lamperti^9^, Data collection and analysis and draft manuscript review

Diego Foschi^9^, Data collection and analysis and draft manuscript review

Ferdinando Ficari^11^, Local principal investigator, acquisition of data, analysis and interpretation of data, and critical review of the article

Ludovica Vacca^11^, Data collection and analysis and draft manuscript review

Marta Cricchio^11^, Data collection and analysis and draft manuscript review

Francesco Giudici^11^, Data collection and analysis and draft manuscript review

Lucio Selvaggi^4^, Local principal investigator, acquisition of data, analysis and interpretation of data, and critical review of the article

Guido Sciaudone^4^, Data collection and analysis and draft manuscript review

Roberto Peltrini^12^, Data collection and analysis and draft manuscript review

Andrea Manfreda^12^, Data collection and analysis and draft manuscript review

Luigi Bucci^12^, Local principal investigator, acquisition of data, analysis and interpretation of data, and critical review of the article

Raffaele Galleano^13^, Local principal investigator, acquisition of data, analysis and interpretation of data, and critical review of the article

Omar Ghazouani^13^, Data collection and analysis and draft manuscript review

Luigi Zorcolo^14^, Local principal investigator, acquisition of data, analysis and interpretation of data, and critical review of the article

Simona Deidda^14^, Data collection and analysis and draft manuscript review

Angelo Restivo^14^, Data collection and analysis and draft manuscript review

Andrea Braini^15^, Data collection and analysis and draft manuscript review

Francesca Di Candido^16^, Local principal investigator, acquisition of data, analysis and interpretation of data, and critical review of the article

Matteo Sacchi^16^, Data collection and analysis and draft manuscript review

Michele Carvello^16^, Data collection and analysis and draft manuscript review

Stefania Martorana^16^, Data collection and analysis and draft manuscript review

Giovanni Bordignon^17^, Statistical analysis and data collection and analysis

Imerio Angriman^17^, Local principal investigator, acquisition of data, analysis and interpretation of data, and critical review of the article

Angela Variola^18^, Data collection and analysis and draft manuscript review

Giuliano Barugola^18^, Data collection and analysis and draft manuscript review

Mirko Di Ruscio^18^, Data collection and draft manuscript

Marta Tanzanu^5^, Literature review and draft manuscript

Andrea Geccherle^18^, Local principal investigator, acquisition of data, analysis and interpretation of data, and critical review of the article

Francesca Paola Tropeano^19^, Data collection and analysis and draft manuscript review

Gaetano Luglio^19^, Local principal investigator, acquisition of data, analysis and interpretation of data, and critical review of the article

Diego Sasia^20^, Data collection and analysis and draft manuscript review

Marco Migliore^20^, Data collection and analysis and draft manuscript review

Maria Carmela Giuffrida^20^, Local principal investigator, acquisition of data, analysis and interpretation of data, and critical review of the article

Enrico Marrano^21^, Data collection and analysis and draft manuscript review

Gianluigi Moretto^21^, Data collection and analysis and draft manuscript review

Harmony Impellizzeri^21^, Local principal investigator, acquisition of data, analysis and interpretation of data, and critical review of the article

Gaetano Gallo^22^, Local principal investigator, acquisition of data, analysis and interpretation of data, and critical review of the article

Giuseppina Vescio^22^, Data collection and analysis and draft manuscript review

Giuseppe Sammarco^23^, Data collection and analysis and draft manuscript review

Giovanni Terrosu^24^, Data collection and analysis and draft manuscript review

Giacomo Calini^24^, Local principal investigator, acquisition of data, analysis and interpretation of data, and critical review of the article

Andrea Bondurri^25^, Data collection and analysis and draft manuscript review

Anna Maffioli^25^, Local principal investigator, acquisition of data, analysis and interpretation of data, and critical review of the article

Gloria Zaffaroni^25^, Data collection and analysis and draft manuscript review

Andrea Resegotti^26^, Data collection and analysis and draft manuscript review

Massimiliano Mistrangelo^26^, Local principal investigator, acquisition of data, analysis and interpretation of data, and critical review of the article

Marco Ettore Allaix^26^, Data collection and analysis and draft manuscript review

Fiorenzo Botti^27^, Data collection and analysis and draft manuscript review

Matteo Prati^27^, Local principal investigator, acquisition of data, analysis and interpretation of data, and critical review of the article

Luigi Boni^27^, Data collection and analysis and draft manuscript review

Serena Perotti^28^, Data collection and analysis and draft manuscript review

Michela Mineccia^28^, Local principal investigator, acquisition of data, analysis and interpretation of data, and critical review of the article

Antonio Giuliani^29^, Local principal investigator, acquisition of data, analysis and interpretation of data, and critical review of the article

Lucia Romano^29^, Data collection and analysis and draft manuscript review

Giorgio Maria Paolo Graziano^30^, Data collection and analysis and draft manuscript review

Luigi Pugliese^30^, Data collection and analysis and draft manuscript review

Andrea Pietrabissa^30^, Local principal investigator, acquisition of data, analysis and interpretation of data, and critical review of the article

GianGaetano Delaini^21^, Conception and design of the study, critical review of the article, and study steering committee

Antonino Spinelli^16,31^, Conception and design of the study, critical review of the article, and study steering committee

Francesco Selvaggi^4^, Conception and design of the study, critical review of the article, and study steering committee

