# Supplementary material for: Surgical treatment of colonic Crohn’s disease: a national snapshot study
Source: Langenbecks Arch Surg. 2020 Dec 2;406(4):1165–72. doi: 10.1007/s00423-020-02038-z (PMC8208904; doi:10.1007/s00423-020-02038-z)
Supplement: Supplementary file 1 — (DOCX 19 kb) [file 423_2020_2038_MOESM1_ESM.docx]

SICCR Current status of Crohn’s disease surgery collaborative. All investigators to be listed as Pubmed citeble authors.

Valerio Celentano ^1,2,3^ ,Gianluca Pellino ^4^, Matteo Rottoli ^5^ , Gilberto Poggioli ^5^ , Giuseppe Sica ^6^ , Mariano Cesare Giglio ^7^, Michela Campanelli ^6^ , Claudio Coco ^8^ , Gianluca Rizzo ^8^ , Francesco Sionne ^8^ , Francesco Colombo  ^9^ , Gianluca Sampietro ^9^ , Giulia Lamperti ^9^, Diego Foschi ^9^ , Ferdinando Ficari ^10^ , Ludovica Vacca ^10^ , Marta Cricchio ^10^ , Francesco Giudici ^10^ , Lucio Selvaggi ^4^ , Guido Sciaudone ^4^ , Roberto Peltrini ^11^, Andrea Manfreda ^11^, Luigi Bucci ^11^, Raffaele Galleano ^12^ , Omar Ghazouani ^12^, Luigi Zorcolo ^13^ , Simona Deidda ^13^ , Angelo Restivo ^13^, Andrea Braini ^14^ , Francesca Di Candido ^15^, Matteo Sacchi ^15^, Michele Carvello ^15^, Stefania Martorana ^15^, Giovanni Bordignon ^16^, Imerio Angriman ^16^ , Angela Variola ^17^, Mirko Di Ruscio ^17^, Giuliano Barugola ^17^, Andrea Geccherle ^17^, Francesca Paola Tropeano ^18^, Gaetano Luglio ^18^, Marta Tanzanu ^5^, Diego Sasia ^19^, Marco Migliore ^19^, Maria Carmela Giuffrida ^19^, Enrico Marrano ^20^, Gianluigi Moretto ^20^, Harmony Impellizzeri ^20^ , Gaetano Gallo ^21^, Giuseppina Vescio ^21^, Giuseppe Sammarco ^22^, Giovanni Terrosu ^23^, Giacomo Calini ^23^, Andrea Bondurri ^24^, Anna Maffioli MD ^24^, Gloria Zaffaroni ^24^ , Andrea Resegotti ^25^ , Massimiliano Mistrangelo ^25^ , Marco Ettore Allaix ^25^, Fiorenzo Botti ^26^, Matteo Prati ^26^ , Luigi Boni ^26^, Serena Perotti ^27^, Michela Mineccia ^27^, Antonio Giuliani ^28^, Lucia Romano ^28^, Giorgio Maria Paolo Graziano ^29^, Luigi Pugliese ^29^, Andrea Pietrabissa ^29^, GianGaetano Delaini ^20^, Antonino Spinelli^,15,30^ , Francesco Selvaggi ^4^, on behalf of the Italian Society of Colorectal Surgery SICCR.

AFFILIATIONS:

1. Portsmouth Hospitals NHS Trust, Portsmouth, United Kingdom
2. University of Portsmouth, Portsmouth, United Kingdom.
3. Department of Surgery and Cancer, Imperial College, London, United Kingdom.
4. Department of Advanced Medical and Surgical Science, Universita’ degli Studi della Campania Luigi Vanvitelli. Naples, Italy.
5. Surgery of the Alimentary Tract, Sant'Orsola Hospital, Department of Medical and Surgical Sciences, Alma Mater Studiorum University of Bologna, Bologna, Italy
6. Minimally Invasive & Gastro-Intestinal Surgical Unit, Department of Surgery, Policlinico Tor Vergata, Rome, Italy
7. Department of Clinical Medicine and Surgery, Federico II University of Naples, Naples, Italy
8. U.O.C. Chirurgia Generale 2 - Fondazione Policlinico Universitario “Agostino Gemelli” IRCCS, Università Cattolica del Sacro Cuore - Rome, Italy
9. IBD Surgical Unit,  Luigi Sacco University Hospital, Milan, Italy
10. IBD Unit, Careggi University Hospital, Florence, Italy
11. University of Naples Federico II, Naples, Italy
12. Santa Corona Hospital, Pietra Ligure, Italy
13. Colon and Rectal Surgery Unit, University of Cagliari, Italy
14. Friuli Occidentale Hospital, Pordenone, Italy.
15. Humanitas Clinical and Research Center IRCCS, Division of Colon and Rectal Surgery, Via Manzoni 56 20089 Rozzano, Milano – Italy
16. Surgical Unit, Department of Surgical Oncological and Gastroenterological Sciences University of Padova. Padova, Italy.
17. IRCCS "Sacro Cuore - Don Calabria" , Negrar (Vr), Italy
18. Department of Public Health, University of Naples Federico II, Naples, Italy
19. Department of Surgery. Santa Croce e Carle Hospital, Cuneo, Italy.
20. Department of Surgery, "Pederzoli" Hospital, Peschiera del Garda, Verona, Italy
21. Department of Medical and Surgical Sciences, University of Catanzaro, Catanzaro, Italy
22. Department of Health sciences, University of Catanzaro, Catanzaro, Italy.
23. Department of Surgery - University Hospital “Santa Maria della Misericordia” - Udine – Italy
24. Unit 1, General Surgery, Luigi Sacco University Hospital, Milan, Italy
25. Department of Surgical Sciences, Citta della Salute e della Scienza di Torino, Presidio Molinette, University Hospital, Turin, Italy.
26. Department of General Surgery, Fondazione IRCCS Ca' Granda Ospedale Maggiore Policlinico, Milan, Italy
27. Division of General and Oncologic Surgery, Mauriziano Hospital, Turin, Italy.
28. San Salvatore Hospital. Department of Biotechnological and Applied Clinical Sciences, University of L'Aquila. L’Aquila, Italy.
29. Fondazione IRCCS Policlinico San Matteo di Pavia, Università degli Studi di Pavia, Pavia, Italy.
30. Humanitas University, Department of Biomedical Sciences, Via Rita Levi Montalcini 4, 20090 Pieve Emanuele Milano – Italy
