# Supplementary material for: Surgical treatment of colonic Crohn’s disease: a national snapshot study
Source: Langenbecks Arch Surg. 2020 Dec 2;406(4):1165–72. doi: 10.1007/s00423-020-02038-z (PMC8208904; doi:10.1007/s00423-020-02038-z)
Supplement: Supplementary file 2 — (DOCX 14 kb) [file 423_2020_2038_MOESM2_ESM.docx]

Appendix 2. Statement of contributions

Principal Investigator and Study Lead: Valerio Celentano

Steering Group: Valerio Celentano, Gianluca Pellino, Antonino Spinelli, Francesco Selvaggi.

Writing Group: Valerio Celentano, Gianluca Pellino, Matteo Rottoli , Mariano Cesare Giglio Francesco Colombo , Luigi Bucci , Andrea Braini , Michele Carvello GianGaetano Delaini , Antonino Spinelli^,^ , Francesco Selvaggi

Local Principal Investigators: Gilberto Poggioli , Giuseppe Sica , Claudio Coco ,Gianluca Sampietro , Ferdinando Ficari Lucio Selvaggi Roberto Peltrini Raffaele Galleano Luigi Zorcolo , Francesca Di Candido, Imerio Angriman , Andrea Geccherle, Gaetano Luglio, Maria Carmela Giuffrida, Gaetano Gallo Giovanni Terrosu, Massimiliano Mistrangelo Michela Mineccia, Antonio Giuliani,

Local Collaborator: Michela Campanelli ,Gianluca Rizzo , Francesco Sionne , Giulia Lamperti, Diego Foschi Ludovica Vacca , Marta Cricchio , Francesco Giudici , Guido Sciaudone Andrea Manfreda Omar Ghazouani , Simona Deidda , Angelo Restivo , Matteo Sacchi , Stefania Martorana , Giovanni Bordignon , Angela Variola , Mirko Di Ruscio , Giuliano Barugola , Francesca Paola Tropeano, Marta Tanzanu , Diego Sasia , Marco Migliore , Enrico Marrano, Gianluigi Moretto , Harmony Impellizzeri Giuseppina Vescio , Giuseppe Sammarco, Giacomo Calini,Andrea Bondurri , Anna Maffioli MD , Gloria Zaffaroni , Andrea Resegotti, Marco Ettore Allaix, Fiorenzo Botti, Matteo Prati , Luigi Boni , Serena Perotti, , Lucia Romano, Giorgio Maria Paolo Graziano, Luigi Pugliese , Andrea Pietrabissa,
